# Macon Medical Society

**Published:** 1885-06

**Authors:** 


### MACON MEDICAL SOCIETY.

Reported for the ATLANTA MEDICAL AND SURGICAL JOURNAL.

STATED MEETING, May 5, 1885.

Dr. J. P. Stevens in the chair.

Dr. Stevens read a paper on

### RHEUMATISM.

He said:—Many years ago Prof. I. K. Mitchell advocated the neurotic origin of rheumatism, and this theory has been more elaborately investigated and still further strengthened by his son, Dr. S. Wier Mitchell. The latter gentleman gives many examples of inflammation and swelling of the joints, strongly similuting arthritic rheumatic inflammations, resulting from nerve and spinal injuries. Carrying out this line of thought, Dr. R. Bartholow, two or three years since, published a very interesting article in the *Medical Record*, wherein three distinct types of rheumatism are recognized, each requiring separate and distinct lines of treatment.

*First.*—Persons of spare habit of body, muscular, and having hereditary tendencies to nervous affections. They are liable to free indulgence in high living, such as rich soups and nitrogenous diet of stimulating and nutritious character, inducing blood plethora. They are also prone to sedentary habits of confinement to in-door employments.

In such patients the salycilate of soda in full doses seems to be specially efficacious, and when this treatment is associated with complete abstinence from a stimulating and nitrogenous diet, substituting therefor starchy and farinaceous substances, peptonized milk, animal broths, etc., the attack is often cut short in less than a week. The tendency, however, is to a relapse, and the treatment should be continued for several days after the subsidence of the fever, pain and swelling, but with a diminished quantity of the salycilates.

*Second.*—Persons of full plethoric habit, obese, and addicted to malt liquors and free living, and subject to acid indigestion. This type seems to be greatly benefited with the alkaline treatment. One and a half ounces of the carbonate is administered in the twenty-four hours—say two drachms every three or four hours in effervescence with lemon juice, or a solution of citric acid. If the bowels are torpid, two or more compound cathartic pills should be given at bedtime to restore the imperfect hepatic secretions. This treatment should be continued as long as the urine shows an acid reaction, when the dose of the alkali should be diminished by one-half—six drachms in the twenty-four hours. After the alkalinity of the urine is overcome, the treatment should be continued with the compound tincture cinchona in a solution of bicarbonate of potash or soda, or three grains of quinine dissolved in a solution of citric acid and half drachm of bicarbonate soda in effervescence three times a day. It is claimed for this method of treatment that the tendency to heart complications is greatly diminished and the relief from suffering is more speedy and the duration of the attack greatly curtailed. According to Dr. Fuller, the average duration, in a tabulated statement of ninety-four consecutive cases, from the commencement of the treatment, was eleven days. The number of heart complications by this method was 4.3 per cent., while that by venesection was 50 per cent. The same precautions as regards the diet should be observed in this as in the former type.

The third type of rheumatic cases is the anæmic subject, characterized by pallor of the skin, looseness and flabbiness of the muscles, and the joints prominent and lax. Such subjects are generally under the influence of bad hygienic surroundings and low and unwholsome diet. As would be legitimately inferrred, the depressing effect of the salycilate and the alkaline treatment is to be feared. In such cases the remedy par excellence is tinct. m[illegible]

ferri. in full doses ʒss. to ʒi. in sufficient water every three or four hours, or, at longer intervals, according to the urgency of the symptoms. Dr. Russell says: "It lessens the swelling and pain of the joints, lowers the fever, diminishes the tendency to heart complications, and above all sustains the vital powers in their struggle against the encroachments of rheumatic disease."

The special efficacy of the blister treatment in association with the before-mentioned remedies cannot be ignored. I have often witnessed the most decided relief from their application to the inflamed joint; so much so, that the patient will often earnestly call for their re-application. It is claimed that blistering induces an alkaline condition of the urine, and they should be allowed to remain on until abundant serosity is accomplished, or, in popular language, until "they bladder" copiously. I am of the opinion that opposition to this remedial agent is based more upon prejudice than upon any well-founded experience of their inefficiency, when judiciously employed.

Dr. Ferguson: Do they claim to cut short the disease in each case?

Dr. Stevens: Yes, in from seven to ten days.

Dr. Gibson: Bartholow recommends the same plan of treatment without the blisters.

Dr. Ferguson: I have never derived any benefit from blisters; it is simply an addition of insult to injury. Neither have I had any benefit from the alkaline treatment, excepting when it was preceded by a good mercurial purge. I cannot see the use of dividing the disease into so many fine types. The benefit from the use of the tinct. of iron is derived from the nitro-muriatic acid, and not from the iron. Local applications have been of no use in my hands; my dependence is entirely on constitutional treatment, and I rely on mercurials in all cases, even if they are anæmic.

Dr. Stevens: I have derived direct and positive benefit from blisters—they certainly relieve pain. I cover the joint entirely and deplete the part—they also have a direct influence on the urine. I have seen every other remedy fail when the blister relieved.

Dr. McHatton: The etyology of this disease (theories excepted) is, as far as I know, unknown. The intrinsic tendency is to recovery. My treatment is at present purely symptomatic in the end, although I start out in each case either on salycilates or the alkalies. My experience in limiting the disease to a period of from six to ten days is, simply that I don't do it. Dr. Stevens, did you ever see, under any plan of treatment, a patient comfortable in seven days?

Dr. Stevens: I never did.

Dr. Gibson: I have a patient that has taken from four to five ounces of salacylic acid every year for several years to prevent the return of the disease, but it always returns.

Dr. Blackshear: I have derived no benefit from medicines. I am inclined to the opinion that vapor baths would be a good plan of treatment, but have had no opportunity to try them.

---

VACCINATION AGAINST CHOLERA.—Dr. Ferman and his assistant, Dr. Pauli, two Spanish physicians of the highest standing, and undoubted authorities on practical microbiology, surprised the meeting of the Academia Medico Quirurgica of last December by the following telegraphic message: "New observations of morphological changes of the bacillus choleræ. Solution of the problem of successfully vaccinating animals. Man tolerates bacsllus-vaccination, as proven by experiments on ourselves." A similar telegram was sent to Professor Letamendi, and next day, as the *El Siglo Medico*, (December 14, 1884, Madrid) states, the secular press brought detailed accounts of this apparently very important discovery. The truth of the self-vaccination and the authority of the observers being apparently unquestionable, it would actually appear as if a new and powerful weapon, i. e., prophylactic vaccination, had at last been found against the choleraic scourge. It is, of course, not yet warrantable to indulge in sanguine anticipations before some more definite and conclusive data are in our possession.—*Therapeutic Gazette.*

